# Supplementary material for: Measuring femoral neck loads in healthy young and older adults during stair ascent and descent
Source: PLoS One. 2021 Jan 26;16(1):e0245658. doi: 10.1371/journal.pone.0245658 (PMC7837459; doi:10.1371/journal.pone.0245658)
Supplement: S1 Appendix — (DOCX) [file pone.0245658.s001.docx]

**Appendi****x A**

**
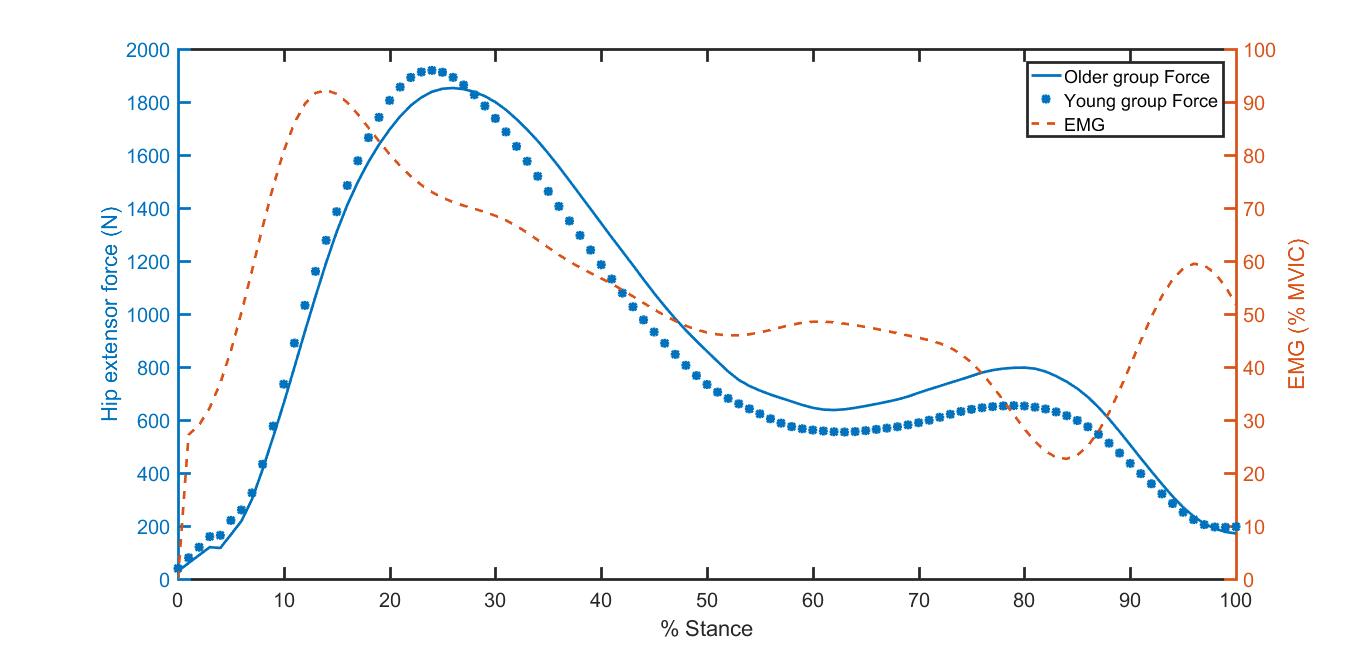
**

**A-1.** Average of estimated hip extensor muscle forces (in Newtons) and EMG activities (in % MVIC) during stair ascent.

**
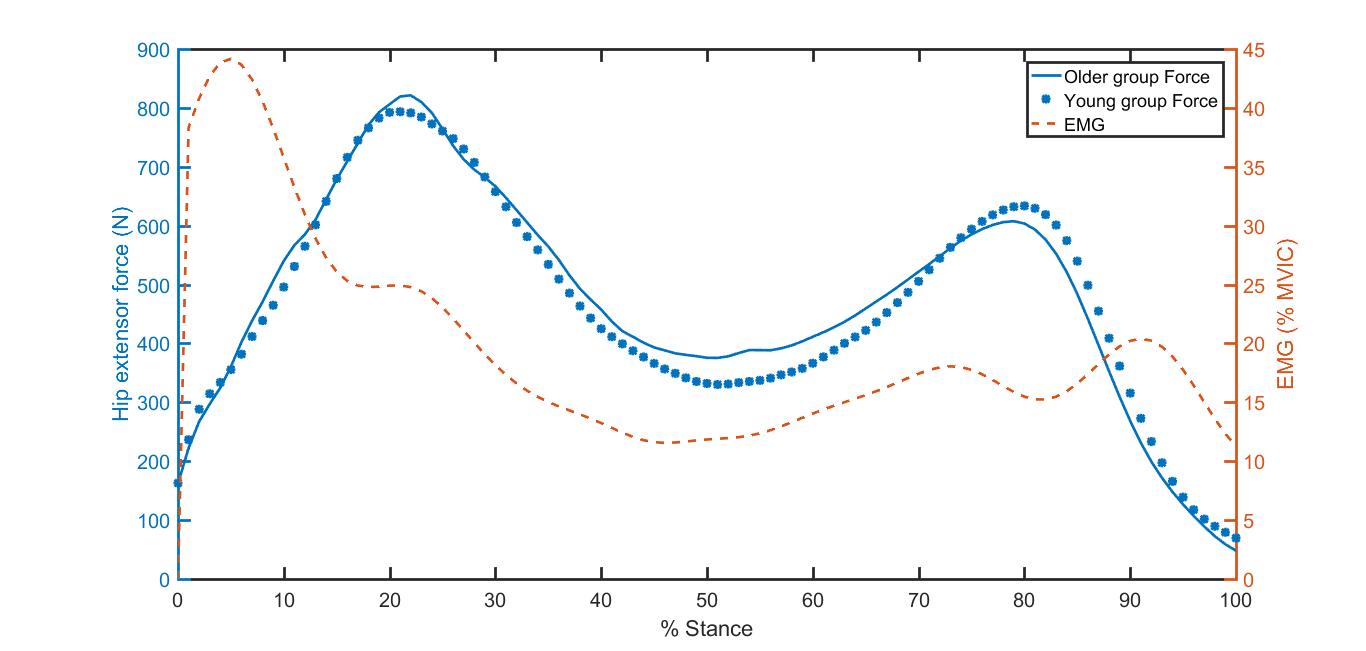
**

**A-2.** Average of estimated hip extensor muscle forces (in Newtons) and EMG activities (in % MVIC) during stair descent.
