# Supplementary material for: Measuring femoral neck loads in healthy young and older adults during stair ascent and descent
Source: PLoS One. 2021 Jan 26;16(1):e0245658. doi: 10.1371/journal.pone.0245658 (PMC7837459; doi:10.1371/journal.pone.0245658)
Supplement: Attachment — Submitted filename: Rebuttal letter to reviewers comments.docx [file pone.0245658.s004.docx]

Hi, there,

As authors, we appreciate all the work the reviewers and editor have done to help us improve the quality of this paper for the second round. Here is the rebuttal letter to the reviewers which replies/answers all the questions and concerns.

Best regards,

Authors for this manuscript

**To reviewer #1:**
General comments:

1.The authors have addressed many of the comments adequately. The reviewers only concern is on the percentage contribution table. It is unclear how >100% contribution could occur. It would be assumed that the total muscle/reaction stress would be 100% or, for example, the muscle force source would add to 100% where each stress site is a contribution of this 100%. Further clarification on how this was calculated is necessary.
Reply: Sorry for the unclear description for the percentage calculation.

In the stress calculation, we calculated the total stress on four sites of the femoral neck separately: superior, inferior, anterior and posterior. For each site, we divided the total stress into several contributions: muscle axial force (along the long axis of the femoral neck), muscle moment (bending effect to the femoral neck), joint reaction force (along the long axis of the femoral neck), and reaction moment (bending effect to the femoral neck).

The percentage calculation was shown as $Percentage=\frac{Contribution}{Totalstress}\times100\%$ (**line 233**). The total stress could be the total stress on any one site we defined above, for example, superior site. Then the contribution could be muscle force/moment, reaction force/moment, or the total contribution from muscle or reaction on this site.

The tensile stress was defined as positive and compressive stress was negative. The stress contributions from muscle/reaction or force/moment could be either tensile (positive) or compressive (negative), the total stress was the sum of stress contributions (Table 2). When these stress contributions contain both tensile and compressive stresses (the compensation effect from different contributions could be found in this case), the total stress could be smaller than one or more stress contributions.

If one contribution (e.g muscle axial force contribution) was greater than the total stress, values >100% could be found in the analysis. Moreover, the sign of the contribution and total stress could be opposite (Table 2), so values like -XX% could be found in the Table 3.

Table 3 was revised and more description was added in **line 234-237, line 250-252**.
